# Supplementary material for: Zn2+ and Cu2+ Interaction with the Recognition Interface of ACE2 for SARS-CoV-2 Spike Protein
Source: Int J Mol Sci. 2023 May 24;24(11):9202. doi: 10.3390/ijms24119202 (PMC10252707; doi:10.3390/ijms24119202)
Supplement: Supplementary file 1 [file ijms-24-09202-s001.zip › ijms-2405419-supplementary.pdf]

Zn^{2+} and Cu^{2+} Interaction with the Recognition Interface of ACE2 for SARS-CoV-2 Spike Protein

Alessio Pelucelli [1], Massimiliano Peana [1,*], Bartosz Orzeł [2], Karolina Piasta [2], Elzbieta Gumienna-Kontecka [2], Serenella Medici [1] and Maria Antonietta Zoroddu [1]

1 Department of Chemical, Physical, Mathematical and Natural Sciences, University of Sassari, 07100 Sassari, Italy; alessiopelucelli@gmail.com (A.P.); sere@uniss.it (S.M.); zoroddu@uniss.it (M.A.Z.)
2 Faculty of Chemistry, University of Wroclaw, 50-383 Wroclaw, Poland; bartosz.orzel@chem.uni.wroc.pl (B.O.); elzbieta.gumienna-kontecka@chem.uni.wroc.pl (E.G.-K.)
* Correspondence: peana@uniss.it

Supplementary Materials

Table S1. Hydrolysis constants for Cu(II) and Zn(II) ions for 0.1M ionic strength, T= 25°C.

**Species**	**logβ**
$Cu(OH)^{+}$	-7.86
$Cu(OH_2)$	-16.47
$Cu(OH_3)^{-}$	-26.65
$Cu(OH_4)^{2-}$	--39.29
$Zn(OH)^{+}$	-9,12
$Zn(OH_2)$	-18,08
$Zn(OH_3)^{-}$	-27,97
$Zn(OH_4)^{2-}$	-39,50

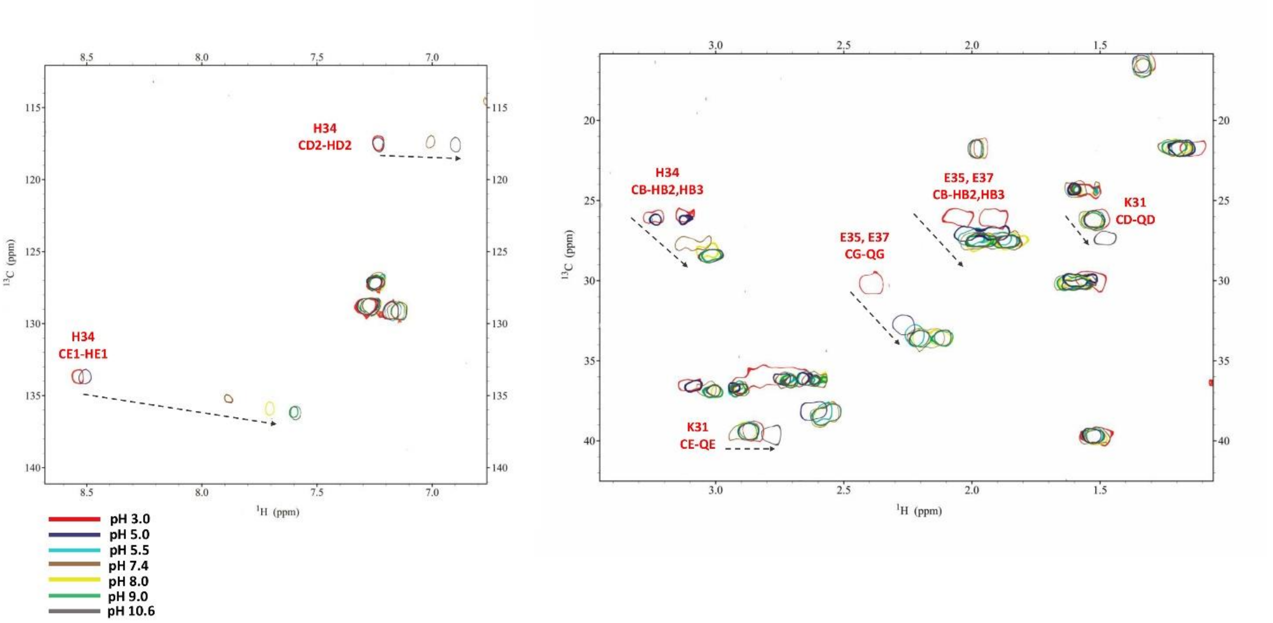

Figure S1. Superimposition of selected region of the ^{1}H-^{13}C HSQC spectra for the free peptide P29-38 at various pH values.

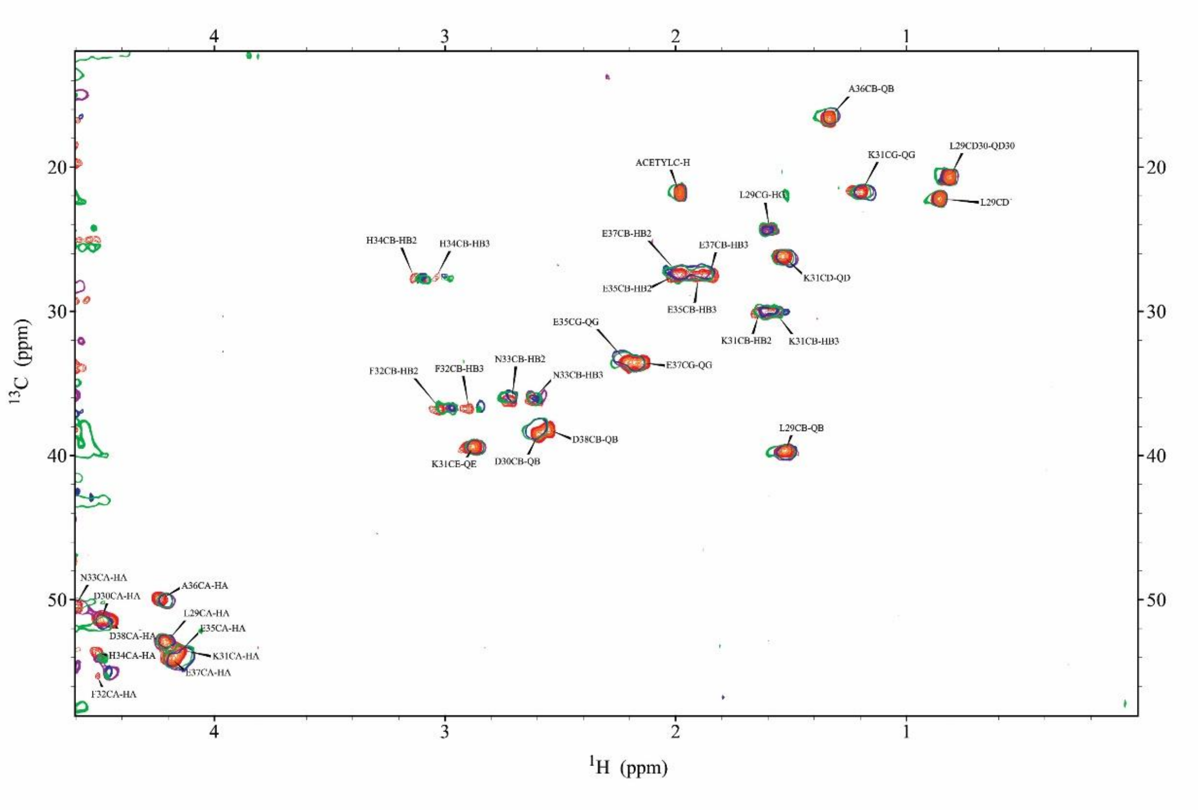

Figure S2. Superimposition of the aliphatic region of the ^{1}H-^{13}C HSQC spectra for the free peptide P29-38 at pH 8 (orange) and the Zn(II)-P29-38 system at pH 8 (blue contours) and pH 9 (green contours).

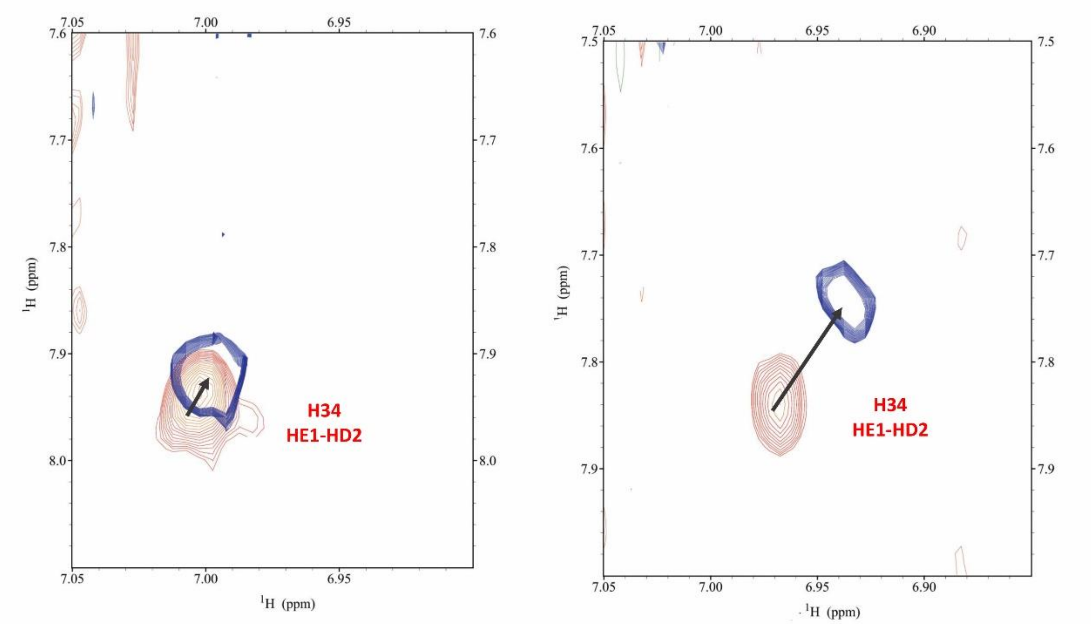

Figure S3. Superimposition of the selected region of the ^{1}H-^{1}H TOCSY spectra for the free peptide P23-42 (orange) and the Zn(II)-P29-38 system (blue contours) at pH 7 (left) and pH 7.5 (right), related to the correlation signals of aromatic protons HE1 and HD2 of His34.

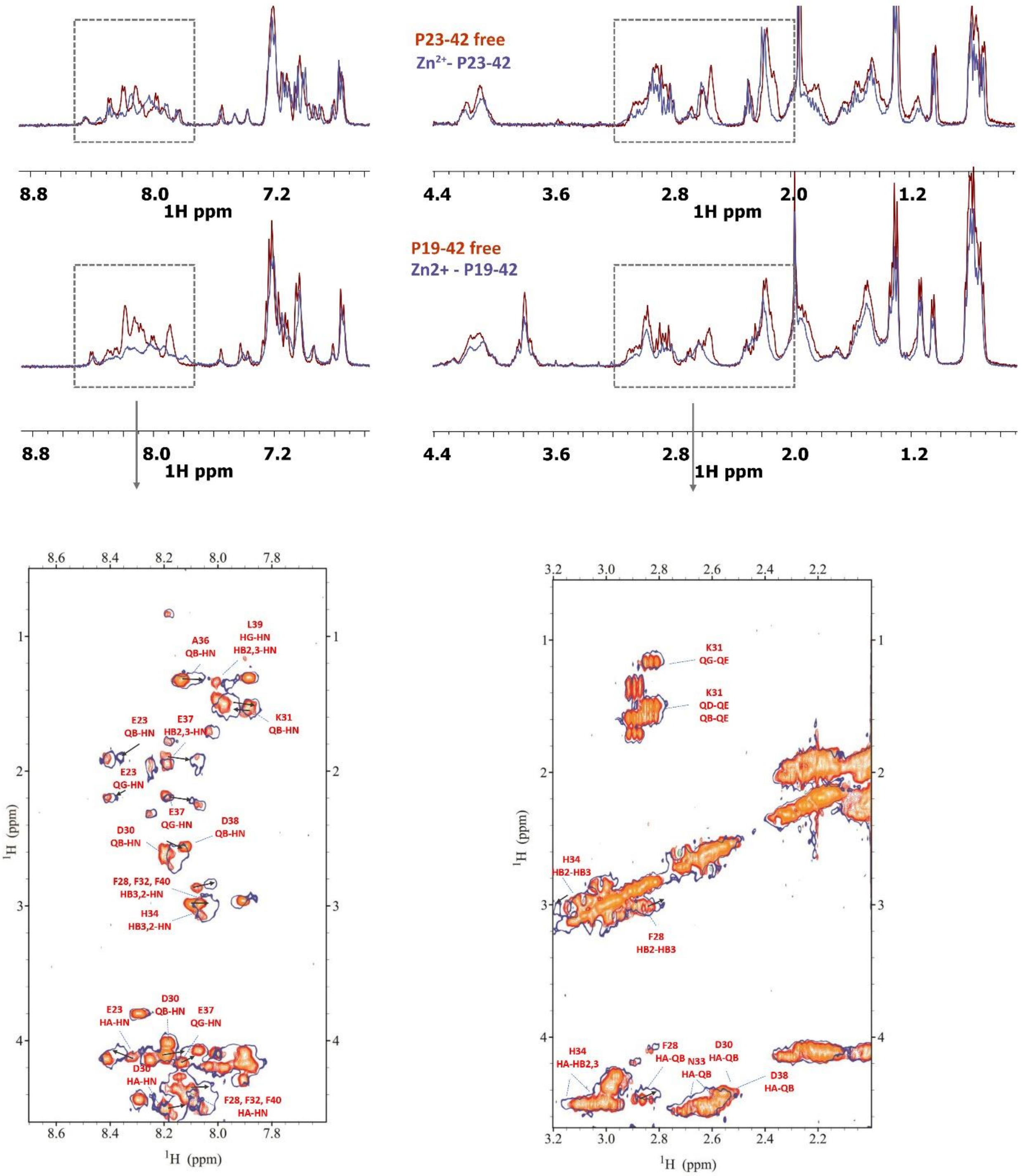

Figure S4. (up) Comparison of ^{1}H spectra for the free peptide P23-42 (orange) and Zn(II)-P23-42 system (blue) and P19-42 (orange) and Zn(II)-P19-42 system (blue) at pH 7.0; (down) selection of ^{1}H-^{1}H TOCSY spectra for the free peptide P19-42 (orange) and Zn(II)-P19-42 system (blue) at pH 7.0.

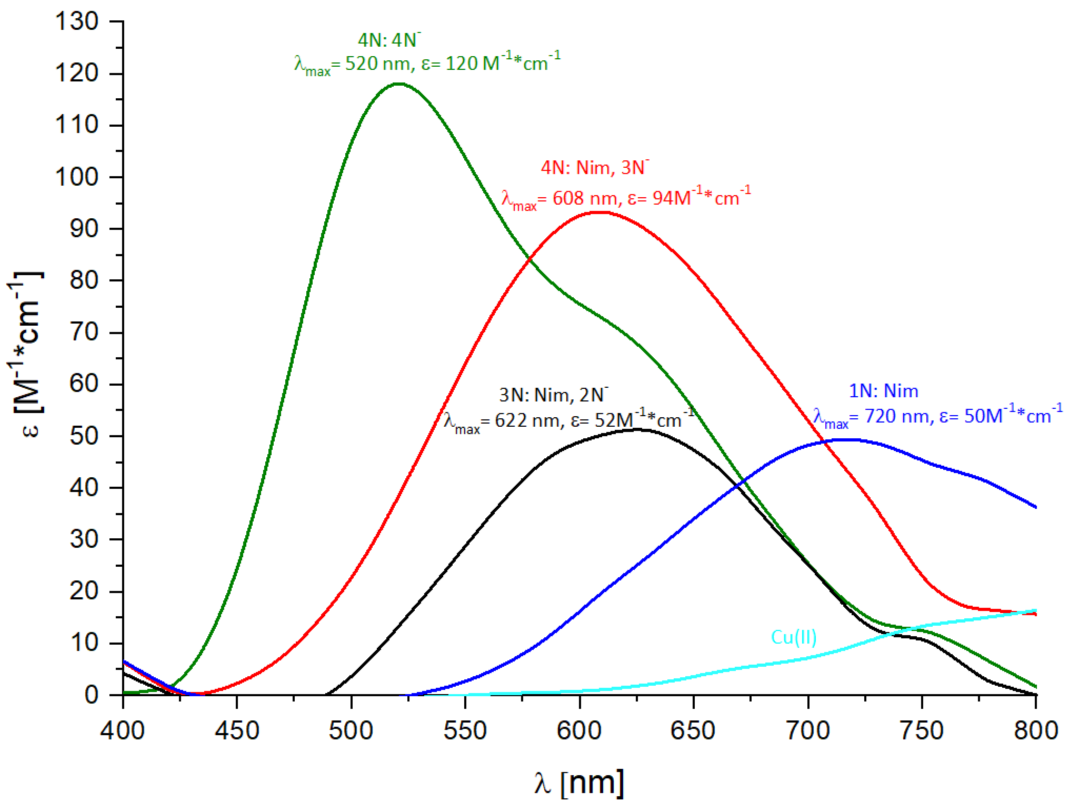

Figure S5. Calculated UV-Vis bands for various Cu(II) complex forms. Bands calculated and drawn by SPECFIT/32 software. cyano: free Cu(II), blue: $[CuHL]^{-}$, black: $[CuLH_{-1}]^{3-}$, red: $[CuLH_{-2}]^{4-}$, green: $[CuLH_{-3}]^{5-}$.

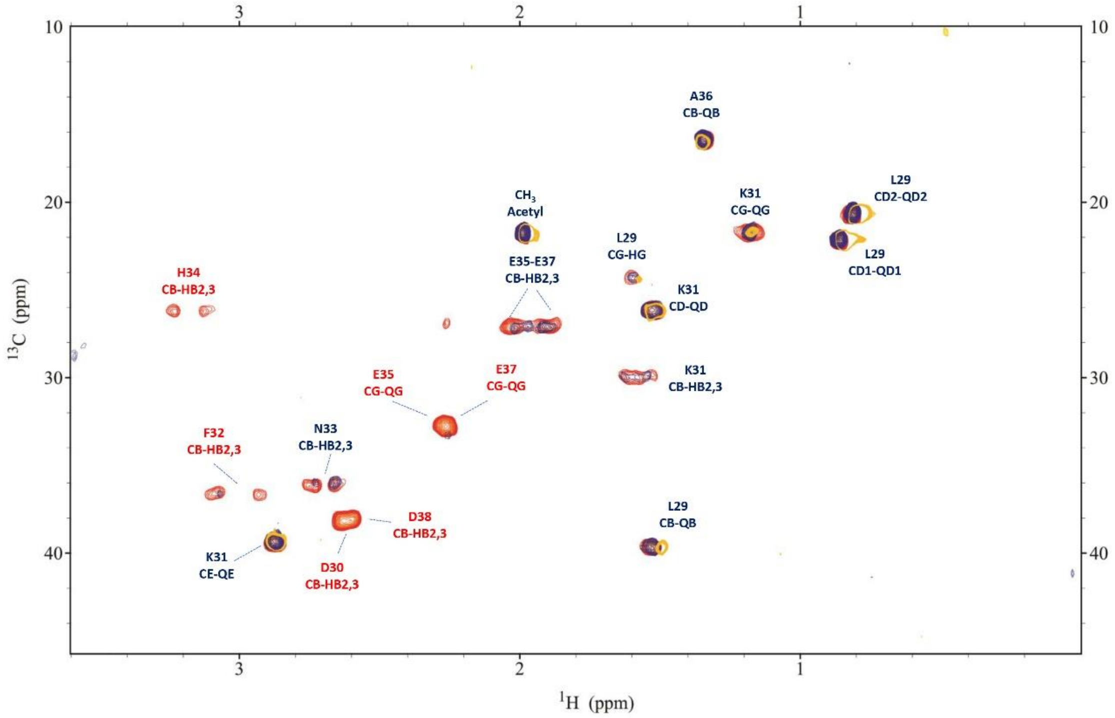

Figure S6. (up) Comparison of a selection aliphatic region of ^{1}H-^{13}C HSQC spectra for the free peptide P29-38 (orange) and Cu(II)-P29-38 system in the molar ratio of 0.01:1 (blue) and 0.1:1 (yellow contours) at pH 5.5.

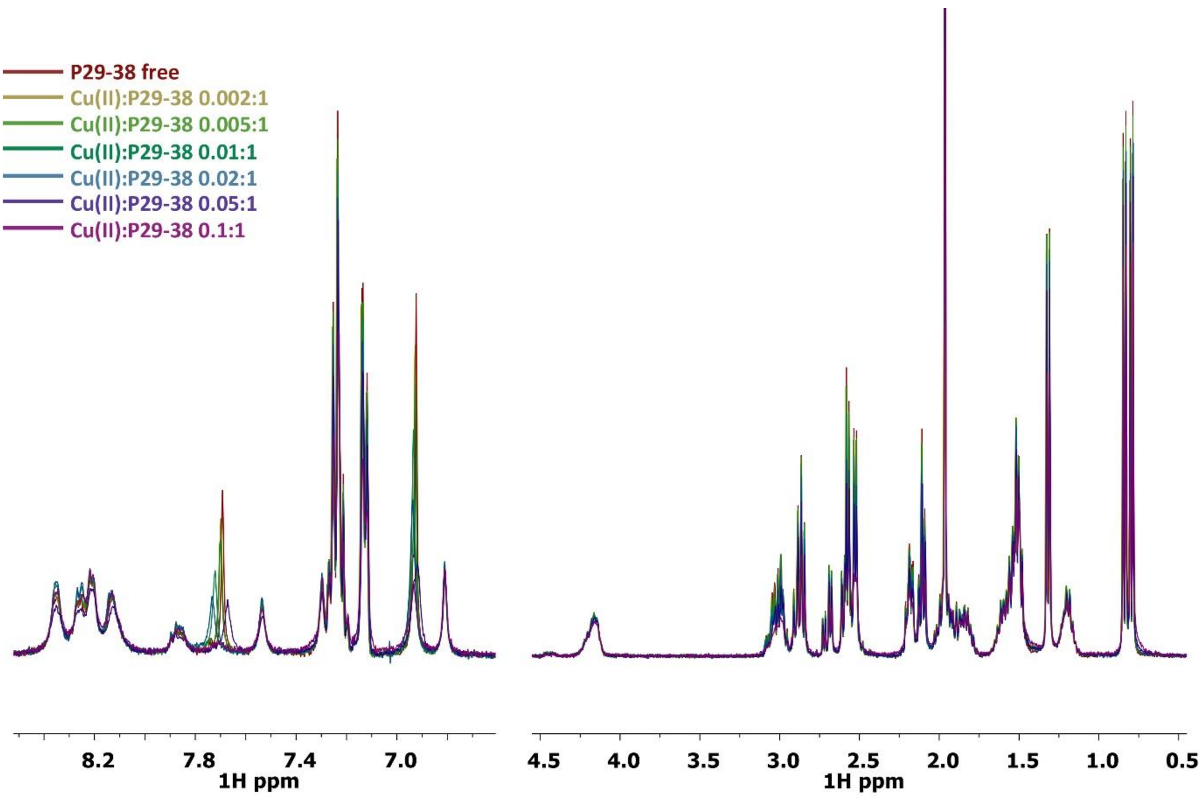

Figure S7. Comparison of ^{1}H spectra of P29-38 with increasing addition of Cu^{2+} at pH 7.4.

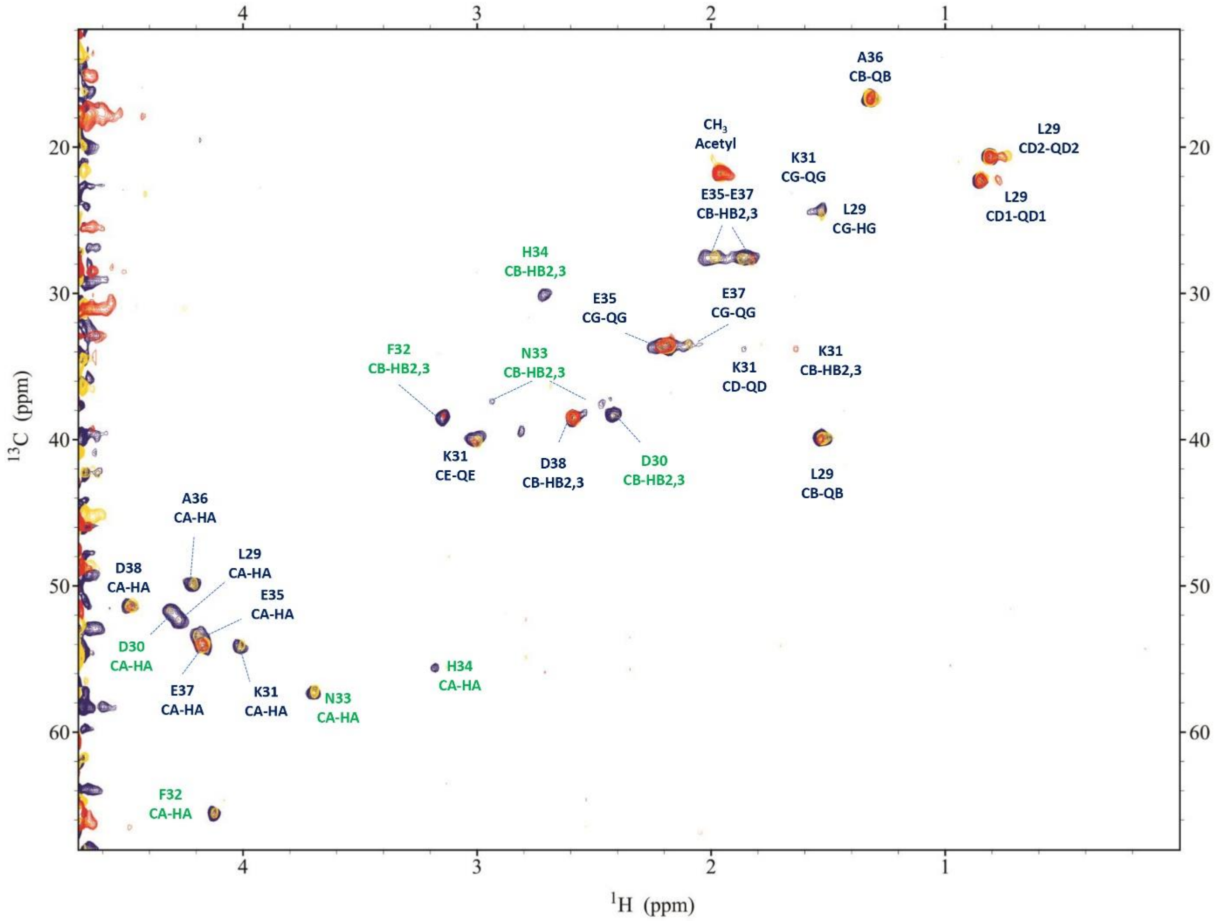

Figure S8. Comparison of aliphatic region of ^{1}H-^{13}C HSQC spectra for the Ni(II)-P29-38 system (blue) with the subsequent addition of 0.4 (yellow) and 0.6 (red) equivalents of Cu(II) at pH 10.6.

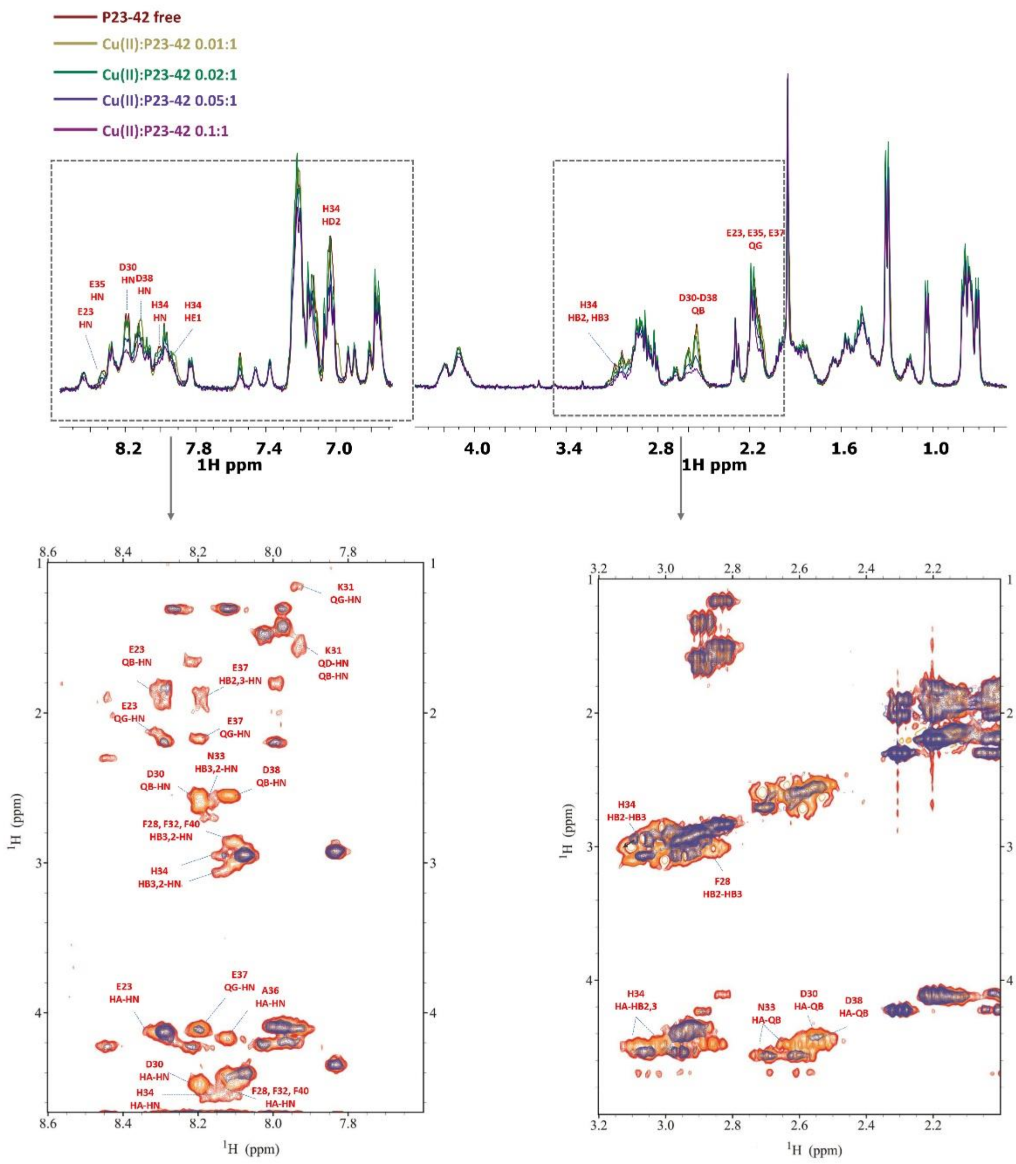

Figure S9. (up) Comparison of ^1H spectra of P23-42 with increasing addition of Cu^{2+} at pH 7.0; (down) selection of ^1H-^1H TOCSY spectra for the free peptide P23-42 (orange) and Cu(II)-P23-42 system (blue), 0.1:1 molar ratio, at pH 7.0.

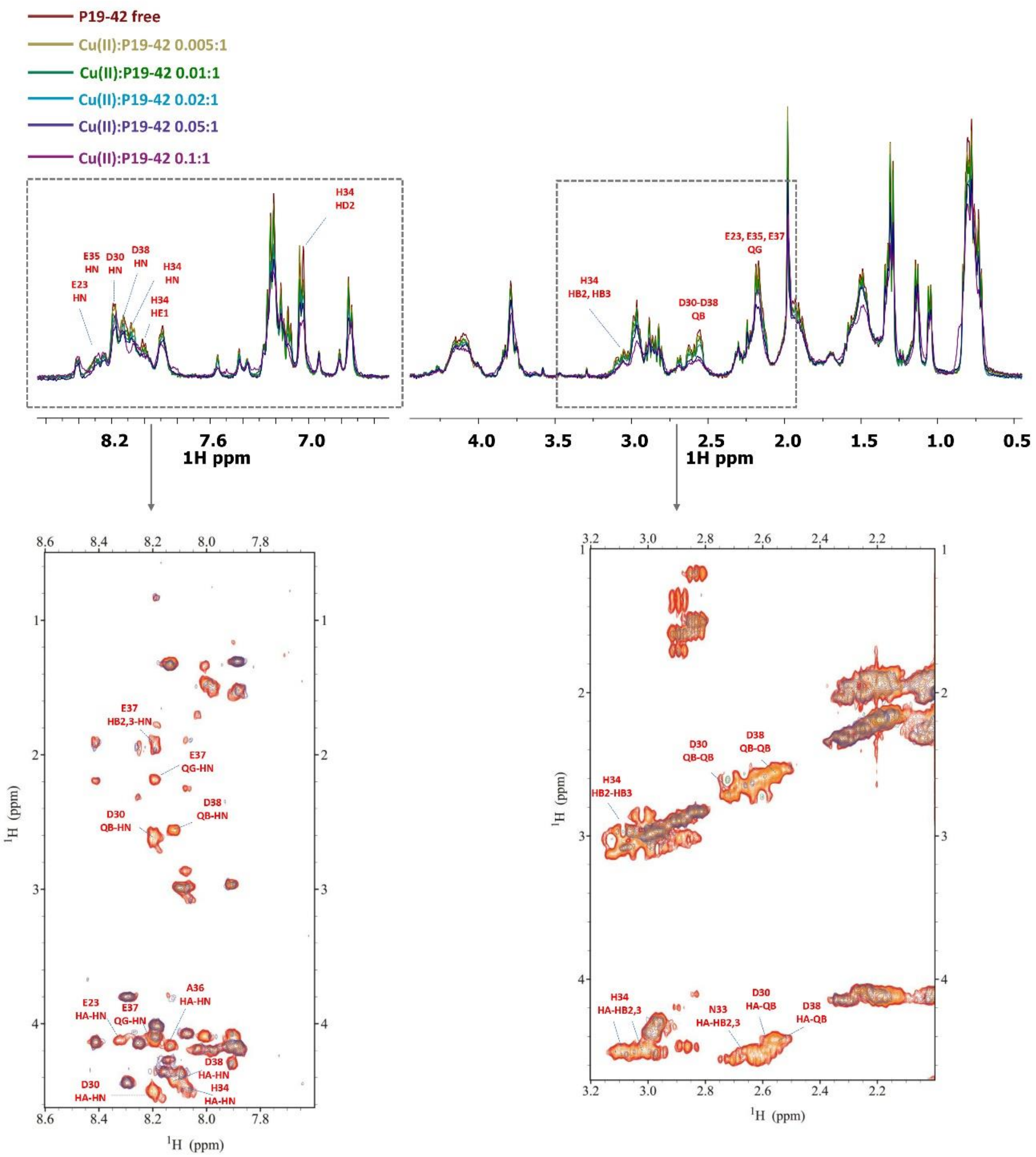

Figure S10. (up) Comparison of ^{1}H spectra of P19-42 with increasing addition of Cu^{2+} at pH 7.0; (down) selection of ^{1}H-^{1}H TOCSY spectra for the free peptide P19-42 (orange) and Cu(II)-P23-42 system (blue), 0.1:1 molar ratio, at pH 7.0.

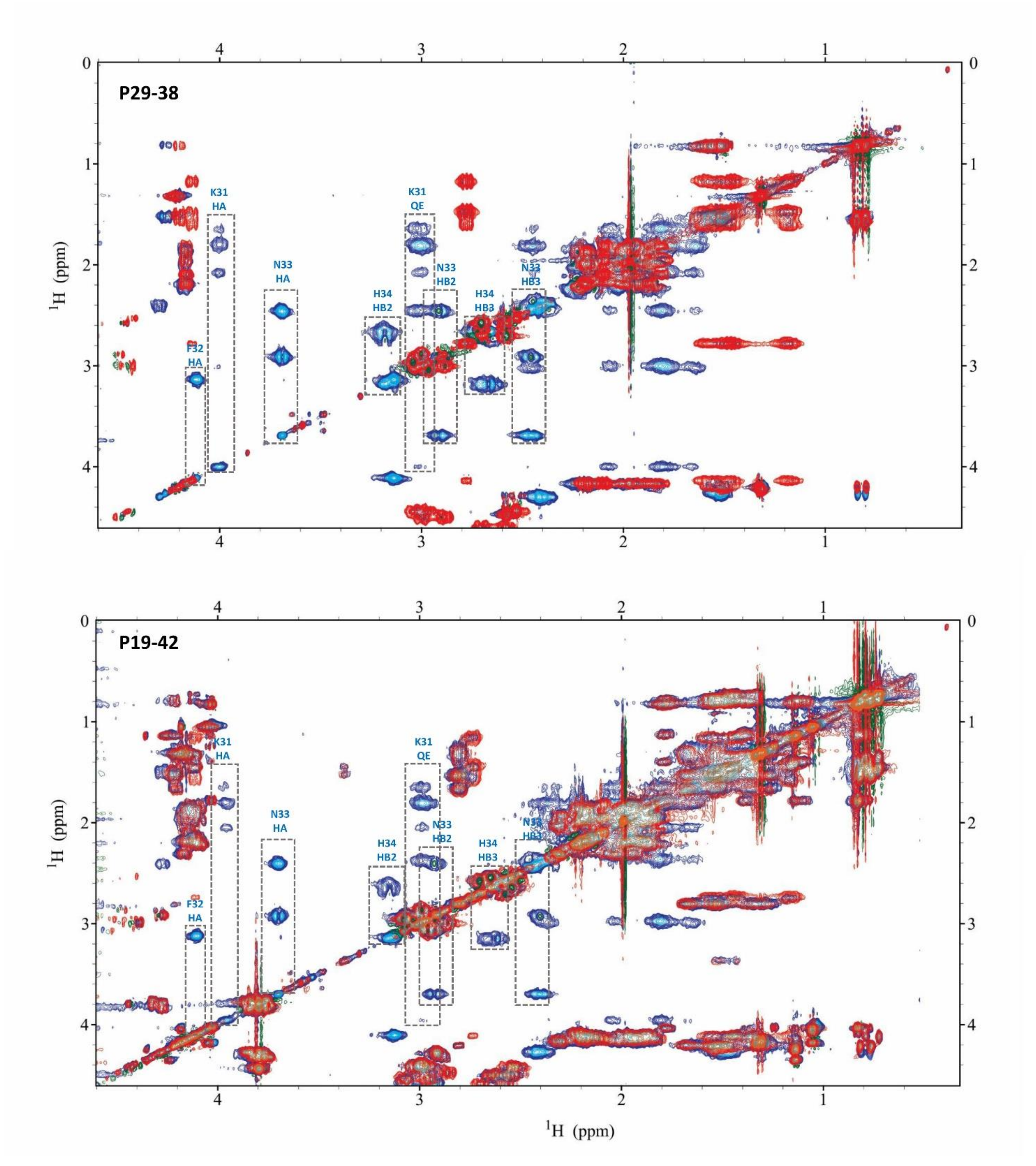

Figure S11. (up) Comparison of aliphatic ^{1}H-^{1}H TOCSY spectra for the free peptide P29-38 (red) and Ni(II)-P29-38 system (blue) and (down) for P19-42 (red) and Ni(II)-P19-42 system (blue) at pH 10.6.

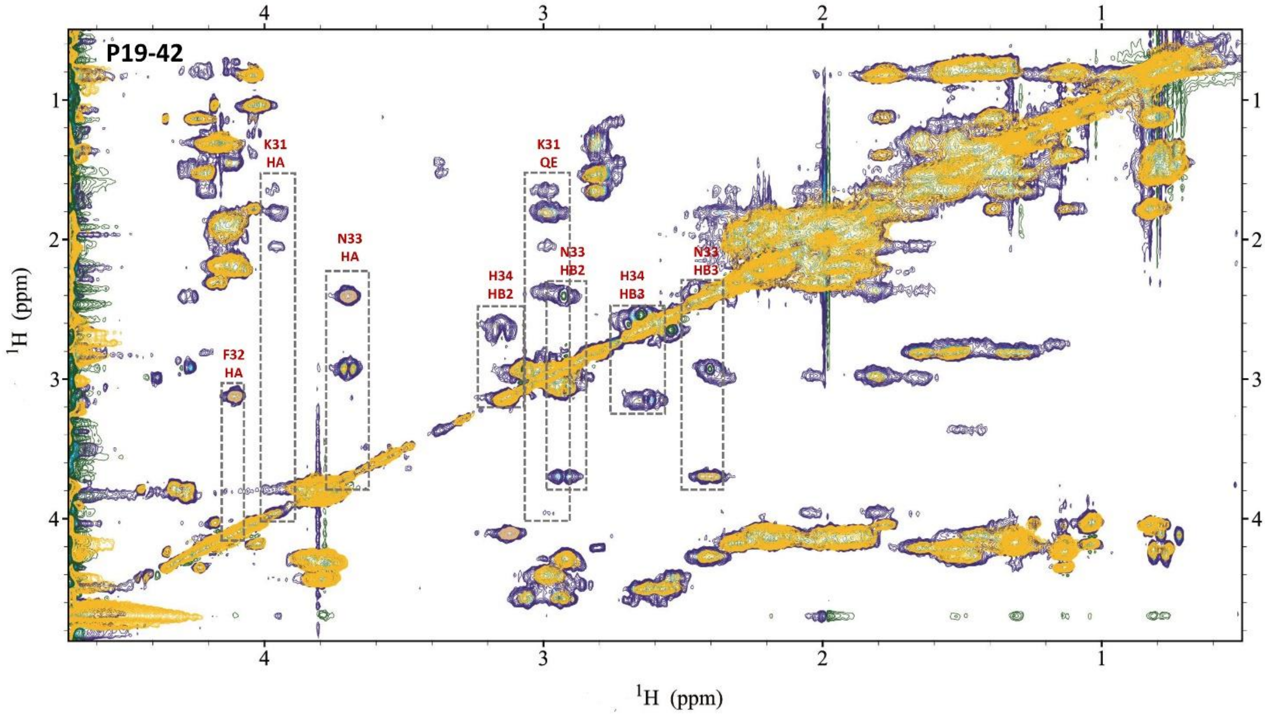

Figure S12. Comparison of aliphatic region of ^{1}H-^{1}H TOCSY spectra for the Ni(II)-P19-42 system (blue) with the addition of 0.6 equivalents of Cu(II) (yellow), at pH 10.6.
